# B7-H3 and Its Role in Antitumor Immunity

**DOI:** 10.1155/2010/683875

**Published:** 2010-11-28

**Authors:** Martin Loos, Dennis M. Hedderich, Helmut Friess, Jörg Kleeff

**Affiliations:** Chirurgische Klinik und Poliklinik, Klinikum Rechts der Isar, Technische Universität München, Ismaninger Straße 22, 81675 München, Germany

## Abstract

B7-H3 is one of the most recently identified members of the B7/CD28 superfamily of costimulatory molecules serving as an accessory modulator of T-cell response. Recently, B7-H3 expression has been reported in several human cancers indicating an additional function of B7-H3 as a regulator of antitumor immunity. However, its precise physiologic role is still elusive, because both stimulatory and inhibitory capacities have been demonstrated. This paper summarizes the available data on B7-H3 in the regulation of T-cell response focusing on its potential role in antitumor immunity.

## 1. Introduction

T lymphocytes of the adaptive immune system are able to recognize and specifically respond to an incredible variety of foreign and native antigens. To ensure an appropriate T-cell response,which is essential to eradicate pathogens and to maintain self-tolerance, T-cell activation is finely tuned by two independent signaling pathways. The first signal requires recognition of the antigen-bearing major histocompatibility complex (MHC) on the surface of antigen-presenting cells (APCs) by the corresponding antigen-specific T-cell receptor (TCR) on T-cells. The second signal, which is antigen independent, is delivered by costimulatory molecules of the B7/CD28 family. B7-1/B7-2:CD28/CTLA-4 signaling represents the best characterized costimulatory pathway [1]. Engagement of B7-1 on APCs with CD28 on T-cells enhances T-cell proliferation and IL-2 production. In the absence of this simultaneous costimulatory signal, ligation of the TCR by an antigenic peptide results in T-cell dysfunction, intolerance or anergy. Apart from stimulatory signals that augment and sustain T-cell responses, costimulatory pathways also deliver inhibitory signals that downregulate or terminate T-cell responses [2]. Binding of CTLA-4 to B7-1 and/or B7-2 inhibits IL-2 synthesis and progression through the cell cycle leading to the termination of T-cell response. Within the past two decades, new costimulatory ligands and receptors have been identified, including B7-H1 (programmed death-1 ligand-1), B7-DC (programmed death-1 ligand-2), PD-1 (programmed death-1), ICOS (inducible costimulator), ICOSL (ICOS-ligand), BTLA (B and T lymphocyte attenuator), B7-H3, and B7-H4 [3].

Recently, these previously identified B7 homologues have been implicated as potential regulators of antitumor immunity. For example, aberrant B7-H1 expression by cancer cells has been associated with adverse pathologic features and poor outcome in different human malignancies and has therefore been postulated as a potential mechanism by which malignant tumors may evade host immune response [4–8]. Taking advantage of manipulation of costimulatory signaling by cancer cells is comprehensible as T-cells play an important role in antitumor immunity. Under normal conditions, APCs that scavenge tumor cell debris and migrate to lymphoid tissues can interact with CD4^+^ and CD8^+^ T-cells to induce activation of T-cells capable of recognizing tumor-specific or tumor-associated antigens. Thus, downregulation of tumor-specific T-cell responses by abusing inhibitory signaling pathways with induction of T-cell anergy or apoptosis through aberrant tumor B7-H1 expression may represent a possible immune escape mechanism. Hence, immune-based therapies which eliminate inhibitory T-cell signaling may represent a potent new approach for the treatment of human malignancies. Indeed, several phase I/II trials using humanized monoclonal antibodies (mAbs) to block CTLA-4 signaling have shown promising results in different human cancers [9–12]. These studies provide evidence that treatment with anti-CTLA-4 mAbs is generally well tolerated and capable of inducing objective tumor responses in patients with prostate cancer, renal cell carcinoma, melanoma, and lymphoma [13–17].

B7-H3 is another recently identified costimulatory molecule that has been implicated as a potential regulator of antitumor response. However, its role in the regulation of T-cell response and in antitumor immunity remains controversial. This paper summarizes the existing data on the immunological function of B7-H3 and focuses on the potential role of B7-H3 in antitumor immunity.

## 2. B7-H3

### 2.1. Structure and Expression Pattern

B7-H3, identified in 2001, is a type I transmembrane protein that shares 20%–27% amino acid identity with other B7 family members [18]. Among the B7 family members, B7-H3 is the most conserved one with ~88% amino acid identity between mice and humans. While murine B7-H3 consists of a single extracellular variable-type immunoglobulin (Ig)V-IgC domain and a signature intracellular domain (2Ig B7-H3), human B7-H3 possesses an additional isoform, the so-called 4Ig B7-H3 that contains a nearly exact tandem duplication of the IgV-IgC domain [19, 20]. The 4Ig transcript is the dominant form in human tissues. So far, only one potential receptor of murine B7-H3 called triggering receptor expressed on myeloid cells (TREM-) like transcript 2 (TLT-2) has been identified. TLT-2 belongs to the TREM receptor family [21]. These receptors function as modulators of cellular responses and play important roles in both innate and adaptive immunities [22]. TLT-2 protein expression has been shown on CD8^+^ T-cells constitutively and is induced on activated CD4^+^ T-cells. Hashiguchi et al. recently found that binding of murine B7-H3 to TLT-2 especially on CD8^+^ T-cells enhances T-cell effector functions such as proliferation, cytokine production, and cytotoxicity. Blockade of the TLT-2:B7-H3 pathway by mAbs against B7-H3 or TLT-2 effectively inhibited both the induction and effector phases of the contact hypersensitivity responses. Although these in vitro data investigating the interaction of murine B7-H3 with TLT-2 suggest that TLT-2 might function as a receptor for B7-H3, Leitner and colleagues did not find evidence for such an interaction in both mice and humans [23]. As an accessory costimulatory molecule, B7-H3 protein is not constitutively expressed on T-cells, natural killer (NK) cells, and APCs, but its expression can be induced on these cell types. In contrast to B7-1 and B7-2 whose expressions are mainly limited to immune cells such as APCs, B7-H3 protein is found on osteoblasts, fibroblasts, fibroblast-like synoviocytes, and epithelial cells as well as in human liver, lung, bladder, testis, prostate, breast, placenta, and lymphoid organs. This broad expression pattern suggests more diverse immunological and probably nonimmunological functions of B7-H3, especially in peripheral tissues. Recently, B7-H3 expression has also been found in a variety of different human cancers, including prostate cancer, clear cell renal cell carcinoma (ccRCC), non-small-cell lung cancer (NSCLC), pancreatic cancer, gastric cancer, ovarian cancer, colorectal cancer (CRC) and urothelial cell carcinoma [24–31]. Although these findings suggest a possible involvement of B7-H3 in the regulation of antitumor immunity, its exact role remains far from clear, because both stimulatory and inhibitory properties have been identified and both beneficial as well as adverse effects of B7-H3 expression in cancers have been reported.

### 2.2. Functional Studies

Initial work on the functional properties of B7-H3 showed a stimulatory effect of human B7-H3 on T-cells. In vitro, B7-H3 was able to increase proliferation of both CD4^+^ and CD8^+^ T-cells, enhance the induction of cytotoxic T lymphocytes (CTLs), and selectively stimulate interferon-gamma (IFN-*γ*) production in the presence of anti-CD3 Abs to mimic the TCR signal [18]. By contrast, inclusion of antisense B7-H3 oligonucleotides decreased the expression of B7-H3 on dendritic cells (DCs) and inhibited IFN-*γ* production by DC-stimulated allogeneic T-cells. Further functional data in support of a stimulatory effect of B7-H3 come from several in vivo studies. Cardiac allografts in treated B7-H3–/– mice showed markedly decreased productions of key cytokine, chemokine, and chemokine receptor mRNA transcripts as compared to wild-type controls [32]. Moreover, the incidence of chronic rejection in two different cardiac allograft models was also inhibited in B7-H3–/– mice as compared to wild-type recipients. In a mouse model of allergic asthma, administration of anti-B7-H3 mAbs significantly reduced airway hyperactivity and resulted in decreased production of Th2 cytokines (interleukin-4 (IL-4), IL-5, and IL-13) as compared with control IgG-treated mice [33]. Furthermore, transfection of B7-H3 into mouse P815 tumor cells that were inoculated into syngeneic DBA/2 mice resulted in complete tumor regression of about one-half of the tumors and amplification of tumor-specific CD8^+^ CTL response [34]. The authors concluded that B7-H3 transfection enhanced the immunogenicity of the inoculated tumor cells. Similarly, injection of a mouse B7-H3 pcDNA3 expression plasmid into EL-4 lymphomas led to complete regression of 50% of tumors or otherwise significantly slowed tumor growth [35]. B7-H3-driven antitumor immunity was mediated by CD8^+^ T-cells and NK cells. In an orthotopic murine colon cancer model, treatment by intratumoral injection of an adenovirus expressing mouse B7-H3 (Ad-B7-H3-GFP) resulted in a reduction of tumor size compared to control animals [36]. In addition, the occurrence of secondary metastasis was significantly reduced. Ad-B7-H3-GFP-treated animals showed significantly higher frequencies of tumor-specific IFN-*γ* producing CD8^+^ T-cells. Based on these mouse cancer models, tumor-associated B7-H3 seems to preferentially regulate CD4-independent induction of CD8^+^ CTL responses. Further lines of evidence for a stimulatory effect of B7-H3 come from a murine hepatocellular carcinoma model. Intratumoral injection of B7-H3-expressing plasmids followed by vasostatin-expressing plasmid injection 24 hours later resulted in a complete eradication of subcutaneous H22 tumors [37]. Interestingly, neither B7-H3 nor vasostatin monotherapy was effective. In contrast to these findings,whichstrongly suggest a stimulatory effect of B7-H3 on T-cell responses and antitumor immunity,other groups have proposed opposite functions for B7-H3. In mice, B7-H3 protein inhibited T-cell activation and effector cytokine production [38]. Furthermore, an antagonistic mAb to B7-H3 enhanced T-cell proliferation in vitro and led to exacerbated experimental autoimmune encephalomyelitis (EAE) in vivo [39]. In B7-H3–/– mice, Th1-mediated hypersensitivity and onset of EAE were promoted, and treatment with a blocking anti-B7-H3 mAb exacerbated EAE [38]. In a murine model of experimental allergic conjunctivitis (EC), administration of anti-B7-H3 mAbs during the induction phase augmented the severity of Th2-mediated EC [40]. In a different study, DC-associated B7-H3 induced by CD4^+^CD25^+^ regulatory T-cells (Tregs) impaired T-cell stimulatory function in vivo [41]. Accordingly, B7-H3–/– mice developed EAE earlier as wild-type littermates. Moreover, B7-H3–/– mice developed more severe airway inflammation under conditions in which T helper cells differentiated toward Th1 rather than Th2. In a different in vitro study, B7-H3 inhibited T-cell proliferation of both CD4^+^ and CD8^+^ T-cells mediated by Ab to T-cell receptor or allogeneic APCs [38].

### 2.3. Retrospective Analyses

In accordance to its inconsistent immunologic function regarding the regulation of T-cell responses which was demonstrated by several in vitro and in vivo studies, the role of B7-H3 in human cancer remains far from clear.

Data in support of a possible stimulatory function of B7-H3 in T-cell and antitumor responses come from retrospective analyses in different human cancers. In gastric cancer, 58.8% of gastric cancer cells in a series of 102 patients have been shown to express B7-H3 in the cell membrane and cytoplasm [26]. Tumor B7-H3 expression positively correlated with survival time, infiltration depth, and tissue type. In pancreatic cancer, high tumor B7-H3 expression by cancer cells in 68 examined patients was significantly associated with prolonged patient survival after surgical resection and significantly correlated with the number of tumor-infiltrating CD8^+^ T-cells [25].

However, several studies in other human cancers correlating tumor B7-H3 expression with clinicopathological features do not concur with these findings. Tumor B7-H3 expression in 70 patients with NSCLC inversely correlated with the number of tumor-infiltrating lymphocytes (TILs) and significantly correlated with lymph node metastasis [24]. In a separate study, the level of circulating soluble B7-H3 (sB7-H3) in patients with NSCLC was associated with higher tumor stage, tumor size, nodal metastasis, and distant metastasis [42]. In ccRCC, 17.4% of tumor cells and 95.1% of tumor vasculature in 743 examined patients expressed B7-H3. B7-H3 expression in either tumor cells or tumor vasculature was found to significantly associate with an increased risk of death from ccRCC [28]. In another clinical study, B7-H3 was found to be uniformly and aberrantly expressed in adenocarcinomas of the prostate (*n* = 338). Marked B7-H3 intensity, which was found in approximately 20% of the examined specimens, was associated with a >4-fold increased risk of cancer progression after surgery [31]. Similar results were shown in another study that investigated B7-H3 expression in 823 patients with prostate cancer. Tumor B7-H3 expression was found in 93% of patients treated with radical prostatectomy [43]. Strong B7-H3 expression in the resected specimens correlated with disease spread and poor outcome. In CRC, strong B7-H3 expression could be observed in 54.3% of 102 CRC patients. Higher B7-H3 expression positively correlated with a more advanced tumor grade and negatively correlated with the intensity of TILs [29]. Most recently, B7-H3 was found to be expressed in 93% of 103 examined ovarian borderline tumors and carcinomas. B7-H3 was also expressed in the endothelium of tumor-associated vasculature in 44% of patients. Carcinomas with B7-H3-positive tumor vasculature were associated with a significantly shorter survival time and a higher incidence of recurrence [27]. All relevant studies regarding the clinical significance of B7-H3 in human cancer are summarized in Table 1 [24–31, 43–47].

### 2.4. Reasons for the Contrasting Immunomodulatory Effects of B7-H3

Based on the latter experimental and clinical data, the role of B7-H3 in human cancer remains unclear. Several explanations for the seemingly conflicting data exist, including the possible existence of additional receptors for B7-H3. So far, only TLT-2 has been identified as a potential receptor for B7-H3 that seems to enhance T-cell effector function in mice [21]. However, B7-H3 might use another receptor besides TLT-2 for its inhibitory function. Furthermore, it has to be considered that most of the existing functional studies have been performed in mouse models and that data regarding the immunomodulatory effects of B7-H3 from these murine studies might not be transferable into humans. Evidence for differences between mouse and human B7-H3 includes the existence of varying isoforms. In mice, the predominant isoform of B7-H3 consists of a classical single extracellular IgV-IgC domain (2Ig B7-H3) whereas the predominant isoform in humans consists of a dual IgV-IgC domain (4Ig B7-H3). It is also conceivable that other additional isoforms may exist. Another possible explanation for the inconclusive data on the functional role of B7-H3 in antitumor response comprises the level of B7-H3 expression on cancer cells that may be of relevance for the induction of different immunological functions. Based on its expression level, B7-H3 may interact with different affinities for several existing receptors and may therefore exert different functions. A similar functional discrepancy regarding the expression levels of a costimulatory molecule in cancer has been shown for B7-1 [48]. Because of inhibitory effects on immune response, low B7-1 expression on murine colon carcinoma cell lines has been implicated as a possible immune-escape mechanism for tumor cells, presumably through binding of the inhibitory T-cell receptor CTLA-4. By contrast, artificially enforced expression of B7-1 on these tumor cells resulted in a strong increase of immunogenicity. The authors of this study concluded that the different effect of B7-1 may be explained by the noticeable higher affinity of B7-1 for CTLA-4 which has been shown to be 100- to 1,000-fold higher than for CD28. Apart from the possible existence of additional B7-H3 receptors, the definition of positive B7-H3 expression and B7-H3 expression levels in the available studies was not standardized. In the study analyzing the role of B7-H3 in human gastric cancer, specimens were scored as B7-H3-expressing tumors when more than 20% of tumor cells stained positive for B7-H3 [26]. Sun et al. defined low tumor-B7-H3 expression in NSCLC when less than 10% of tumors expressed B7-H3 [24]. In this study, 37.1% of the examined specimens expressed B7-H3. In ccRCC, tumors with less than 10% of cells stained positive were scored as having negative B7-H3 expression. 17% of specimens revealed positive tumor cell B7-H3 expression [28]. Zang et al. did not define the difference between high or low tumor B7-H3 expression in prostate cancer at all [43]. In none of these studies, B7-H3 staining intensity was not taken into account. In our recent study, the most detailed scoring system was used for expression analysis of B7-H3 in pancreatic cancer. Scores were given separately for the stained area and for the intensity of staining [25]. In addition to the different definitions of positive B7-H3 expression and B7-H3 expression levels that were used in previous retrospective studies, a possible influence of soluble forms of B7-H3 was not examined. Based on a study analyzing sB7-H3 levels in NSCLC that showed that the level of circulating sB7-H3 was associated with higher tumor stage, tumor size, nodal metastasis, and distant metastasis, one could speculate that sB7-H3 may also contribute to the modulation of immune response [42]. Another possible reason may include the expression of aberrant forms of B7-H3 on tumor cells which cannot be differentiated by the existing antibodies.Yi and Chenrecently worried that many so-called “neutralizing antibodies” may not be just blocking antibodies but have other effects such as triggering the B7-H3 signal [49]. Furthermore, genetic polymorphisms in B7-H3 may modify T-cell responses in human cancers. Recent studies have shown that polymorphisms in the inhibitory molecule CTLA-4 alter cancer susceptibility through modification of T-cell response [50]. Finally, B7-H3 may also affect other immune cells than T-cells. In neuroblastoma, 4Ig-B7-H3 molecules expressed at the tumor cell surface have been shown to exert a protective role from NK-mediated lysis by interacting with a still undefined inhibitory receptor expressed on NK cells [19].

### 2.5. Therapeutic Potential of B7-H3

Immune-based therapies which additionally stimulate T-cell activation or eliminate inhibitory T-cell signaling in order to enforce tumor-reactive T-cell responses represent a potent new approach for the treatment of human malignancies. Blockade of the inhibitory receptor CTLA-4 by mAbs has been tested as a single agent or in combinations in patients with advanced cancer, including breast cancer, lymphoma, melanoma, ovarian cancer, prostate cancer, and ccRCC [9, 15, 17, 51–54]. Most trials have not only shown that anti-CTLA-4 mAb treatment is safe but also provide evidence for its antitumor effects. In unresectable advanced melanoma for instance, durable tumor responses and disease control rates have been observed. PD-1 is another inhibitory receptor expressed on activated T-cells that may suppress antitumor immunity. Therefore, single-agent anti-PD-1 blockade has been tested in a Phase I trial of 39 patients with advanced metastatic melanoma, CRC, NSCLC, castrate-resistant prostate cancer, and ccRCC. Anti-PD-1 treatment resulted in one durable complete response and two partial responses [55].

Given its immunomodulatory capacities, B7-H3 may also represent a new target in cancer treatment. In contrast to CTLA-4 and PD-1, however, one has to take into account that B7-H3 is more broadly expressed, especially in peripheral healthy tissues. Therefore, blockade of B7-H3 by mAbs or treatment with B7-H3 (i.e., by gene transfer) may be associated with severe adverse effects. Furthermore, the functional role of B7-H3 in antitumor immunity is not completely understood, and controversies regarding its stimulatory and inhibitory capacities remain to be elucidated.

## 3. Conclusions

Within the past decade, new insights into immunomodulatory capacities of costimulatory signaling in antitumor response have opened the door for new potent approaches in cancer therapy. Recent clinical Phase I/II trials have provided solid evidence that treatment with anti-CTLA-4 mAbs is capable of inducing objective antitumor responses. B7-H3, a recently identified member of the B7/CD28 superfamily of costimulatory molecules, has been shown to play an important role in immune regulation. Although data on the precise role of B7-H3 in the regulation of T-cell responses and especially in antitumor immunity has yet to be elucidated, B7-H3 represents a promising new target for immune-based antitumor therapies. The previous identification of the costimulatory receptor TLT-2 is the first significant step toward resolving the available conflicting data.

However, further work particularly concerned with the identification of inhibitory receptors for B7-H3 is ongoing. 

## Figures and Tables

**Table 1:** Relevant clinical studies investigating the relationship between B7-H3 expression in human cancer tissues with clinicopathological features.

Author	Journal	Year	Type of malignancy	Number of patients	Positive tumor-associated	Correlation with clinicopathologic features	
					B7-H3 expression	Favorable	Adverse

Sun et al. [29]	Cancer Immunol Immunother	2010	Colorectal cancer	102	87.3%	—	Higher tumor B7-H3 correlated with a more advanced tumor grade

Zang et al. [27]	Mod Pathol	2010	Ovarian carcinoma	103	93% of tumor cells	—	Significant shorter survival time and higher incidence of recurrence for patients with positive B7-H3 tumor vasculature
					44% of tumor vasculature		
Parker et al. [46]	Int J Radiat Oncol Biol Phys	2010	Recurrent prostate cancer	148	100%	—	Increased risk of biochemical recurrence for patients with moderate and marked B7-H3 staining

Loos et al. [25]	BMC Cancer	2009	Pancreatic cancer	68	88.2%	high tumor B7-H3 expression was associated with significantly better postoperative prognosis	—

Boorjian et al. [44]	Urology	2009	Renal angiomyolipoma/ pulmonary lymphangio-leiomyomatosis	110/7	100% and 2.7%	—	—

Yamato et al. [47]	Br J Cancer	2009	Pancreatic cancer	59	93.2%	—	Strong tumor B7-H3 expression was significantly associated with lymph node metastasis and advanced pathological stage

Crispen et al. [28]	Clin Cancer Res	2008	ccRCC	743	17% of tumor cells	—	Either tumor cell or diffuse tumor vasculature B7-H3 expression was significantly associated with an increased risk of death from ccRCC
					95% of tumor vasculature		
Boorjian et al. [30]	Clin Cancer Res	2008	Urothelial cell carcinoma	318	70.7%	—	—

Greorio et al. [45]	Histopathology	2008	Neuroblastoma	53	74%	—	High tumor B7-H3 expression was associated with a worse event-free survival

Zang et al. [43]	PNAS	2007	Prostate cancer	823	93%	—	Patients with strong tumor B7-H3 expression were at significantly increased risk of clinical cancer recurrence and cancer-specific death

Roth et al. [31]	Cancer Res	2007	Prostate cancer	338	100%	—	(1) Increasing levels of tumor B7-H3 intensity correlated with worsening clinicopathologic features, including tumor volume, extraprostatic extension, higher Gleason score, seminal vesicle involvement, surgical margins
							(2) Marked tumor B7-H3 intensity was significantly associated with cancer progression
Sun et al. [24]	Lung Cancer	2006	NSCLC	70	37.1%	—	High tumor B7-H3 expression was significantly more common in cases with lymph node metastasis

Wu et al. [26]	World J Gastroenterol	2006	Gastric cancer	102	58.8%	Positive tumor B7-H3 expression was significantly associated with better postoperative survival	—
